# Diffusible signals from *Azospirillum brasilense* promote rice growth and induce distinct gene expression changes in plant roots

**DOI:** 10.3389/fpls.2026.1911178

**Published:** 2026-10-12

**Authors:** Samuel Hoggard, Matthew Calhoun, Anna Bommes, Yasir Rahmatallah, Dylan Neuhaus, Galina Glazko, Arijit Mukherjee

**Affiliations:** 1 Department of Biology, University of Central Arkansas, Conway, AR, United States; 2 Department of Biomedical Informatics, University of Arkansas for Medical Sciences, Little Rock, AR, United States

**Keywords:** *Azospirillum brasilense*, diffusible signals, plant growth-promoting bacteria, rice, RNA-Seq

## Abstract

Plants form associations with diverse beneficial microbes, including arbuscular mycorrhizal (AM) fungi, rhizobia, and plant growth-promoting bacteria (PGPB). Research on legume-rhizobia symbiosis (LRS) and AM symbiosis has demonstrated that molecular communication between symbiotic partners is essential for initiating these interactions. Genetic and biochemical studies have further identified both plant and microbial signals, as well as host genetic pathways, that regulate these interactions. However, most studies on plant-PGPB interactions have focused on direct application of PGPB to plant roots, leaving the early signaling mechanisms underlying these interactions poorly understood. In this study, we report that diffusible signals from *A. brasilense* can promote rice growth without direct contact between bacteria and plant roots, 7 days post-treatment (dpt). Using RNA sequencing, we identified 2,516 differentially expressed genes (DEGs) in rice roots in response to diffusible signals from *A. brasilense* at 1 dpt. We identified differential expression of genes involved in flavonoid synthesis, defense responses, receptor activity, transcription factors, and hormone pathways. Comparative transcriptomic analysis of rice roots with or without contact with *A. brasilense* revealed both shared and unique gene expression patterns. We observed upregulation of certain defense-response genes (*PAL*, chitinase, thionin), transcription factors (AP2/ERFs, MYBs, WRKYs, and GRAS), and hormone-related (auxin and ethylene) genes without bacterial contact, but downregulation or no differential expression of these genes upon bacterial contact. This pattern suggests that initial induction followed by subsequent suppression of these genes may be crucial for establishing the interaction with *A. brasilense.* Our transcriptomic analysis also suggests that flavonoids may be required throughout the interaction, whereas nitrate transporters appear to be involved at later stages and likely require contact between plant roots and bacteria. Collectively, our findings reveal that diffusible signals from *A. brasilense* can promote rice growth and induce distinct gene expression changes in plant roots. These results provide novel insights into the early phase of rice-*A. brasilense* interaction and highlight the potential for leveraging plant-PGPB signaling mechanisms to enhance crop growth under adverse environmental conditions.

## Introduction

Plants can interact with a range of beneficial microbes, including rhizobia bacteria, arbuscular mycorrhizal (AM) fungi, and plant growth-promoting bacteria (PGPB) (Santi et al., 2013; Mukherjee and Ané, 2011b; Glick, 2012; Vishwakarma et al., 2020). In these mutualistic relationships, host plants benefit from increased growth in return for carbohydrates provided to the microbial partner. Extensive research has elucidated the mechanisms by which these beneficial microbes promote plant growth. For example, in the legume-rhizobia symbiosis (LRS), soil bacteria rhizobia improve host plant growth primarily via atmospheric nitrogen fixation (Oldroyd and Downie, 2008; Roy et al., 2020; Mukherjee and Ané, 2011b). In interactions with PGPB, such as *Azospirillum brasilense* and *Herbaspirillum seropedicae*, plant growth is improved via multiple mechanisms, including nitrogen fixation, hormone synthesis, and protection against biotic and abiotic stresses (Olanrewaju et al., 2017; Glick, 2012; Santi et al., 2013; Backer et al., 2018). Similar to AM fungi, PGPB have a broad host range, encompassing major crops such as rice, corn, and wheat. Consequently, advancing our understanding of plant-microbe interactions remains essential for improving crop yields, particularly under environmental stresses such as salinity and drought.

Multiple decades of biochemical and genetic studies have identified the plant and microbial signals involved in LRS (Oldroyd and Downie, 2008; Roy et al., 2020; Mukherjee and Ané, 2011b). For example, these studies identified an initial signal exchange between the host plant roots and the interacting microbe before microbial colonization of the roots and the subsequent development of root nodules, within which nitrogen fixation occurs. The initial signal exchange begins with plant roots secreting flavonoids into the rhizosphere, which interacting compatible bacteria perceive, leading to the synthesis and secretion of lipochitooligosaccharides or Nodulation factors (Nod factors) (Peters et al., 1986; Dénarié et al., 1996; Denarie and Cullimore, 1993). Plant molecular receptors perceive these rhizobial Nod factors, triggering morphological, physiological, and transcriptomic changes that culminate in root nodule development and maintenance (D'Haeze and Holsters, 2002; Denarie and Cullimore, 1993; Oldroyd and Downie, 2008; Roy et al., 2020). Later studies identified a similar signal exchange between plant roots and AM fungi during their interaction (Kosuta et al., 2003; Navazio et al., 2007; Olah et al., 2005; Mukherjee and Ané, 2011a). The AM fungal signaling molecules were later identified as Myc factors, a mix of chitin oligosaccharides and lipochitooligosaccharides (Maillet et al., 2011). Analogous to Nod factors, Myc factors activate host symbiosis signaling required to establish the plant-AM fungi interaction (Camps et al., 2015; Sun et al., 2015). Numerous studies have identified changes in gene expression in host plants upon application of exogenous Nod and Myc factors (Breakspear et al., 2014; Camps et al., 2015; Jardinaud et al., 2016; Czaja et al., 2012; Liu et al., 2023). In comparison, our understanding of the early signaling mechanisms underlying plant-PGPB interactions remains largely unknown.

The identification of specific plant- and bacterial-derived signals in plant-PGPB interactions indicates a signaling exchange between host plants and PGPB. For example, plant root exudates such as sugars, organic acids, amino acids, and flavonoids serve as chemoattractants, facilitating PGPB colonization (Chen and Liu, 2024; Sasse et al., 2018; Han et al., 2023). Several genes from the flavonoid biosynthesis pathway are differentially expressed in plant roots following inoculation with various PGPB, suggesting a role for flavonoids in these interactions (Wiggins et al., 2022; Thomas et al., 2019; Camilios-Neto et al., 2014; Brusamarello-Santos et al., 2019). Conversely, PGPB release phytohormones, diffusible signals, and volatile organic compounds (VOCs) that may serve as molecular signals influencing plant growth and development (Ryu et al., 2003, 2004; Kanchiswamy et al., 2015; Mendez-Gomez et al., 2021). Several studies have investigated the effects of VOCs on plants, including one that reported how bacterial VOCs led to several metabolic alterations (e.g., including protein synthesis, signaling, and energy metabolism) in rice and induced plant growth (Almeida et al., 2023). Recently, a study examined three interaction modes (direct contact, diffusible signals, or VOCs) between *A. brasilense* and *Arabidopsis thaliana* and reported that the bacterium improved plant mass in all systems tested (Mendez-Gomez et al., 2021). These findings suggest that diffusible signals and VOCs secreted by *A. brasilense* are likely recognized by the plant, leading to its growth promotion. Additionally, the authors reported that *A. brasilense-*secreted molecules promoted plant growth by modulating the auxin and ethylene signaling pathways in *A. thaliana* (Mendez-Gomez et al., 2021). Despite these advances, further research is needed to characterize host growth and molecular responses to growth-inducing signals from PGPB, particularly in crops such as rice. In this study, we investigate rice growth and transcriptomic responses to diffusible signals from *A. brasilense*. We hypothesize that diffusible signals from *A. brasilense* can promote rice growth and induce significant transcriptional changes in plant roots, including changes in the expression of flavonoid synthesis genes, defense-response genes, genes encoding plant receptors and transcription factors, and hormone-related genes. Results from this study will contribute to our understanding of the underlying signaling mechanisms in the rice-*A. brasilense* interaction, which can have significant implications for improving crop growth under adverse environmental conditions.

## Materials and methods

### Plant growth and preparation

Plant growth and preparation procedures used in this study were based on those reported in previous studies (Wiggins et al., 2022; Thomas et al., 2019; Hiltenbrand et al., 2016). After surface-sterilization and germination, rice seedlings were transferred to sterile Petri plates (#639102, Greiner Bio-One, Monroe, NC, USA) containing sterile plant agar supplemented with low-nitrogen Fahraeus Medium (FM). The seedlings were grown for another seven days in a Percival growth chamber (#CU-22L, Perry, IA, USA) with a light intensity of 150-200 µmol m^-2^s^-1^ and a relative humidity of 65%. All work was performed in a sterile laminar flow hood (Thermo Scientific 1300 series A2, Waltham, MA, USA).

### Bacterial treatments: contact and diffusible treatments

*Azospirillum brasilense* Sp245 were grown as described in previous studies (Thomas et al., 2019; Wiggins et al., 2022; Degon et al., 2023). Briefly, *A. brasilense* were grown on Tryptone Yeast-Extract (TY) medium at 30°C to an optical density (600 nm) of 0.6, then resuspended in sterile water and used for inoculation. Sterile water was used as a mock treatment in these experiments. After the initial growth on the Petri plates (#639102, Greiner Bio-One, Monroe, NC, USA) as mentioned earlier, the rice seedlings were transferred to Square Bioassay Dishes (LXWXH, 245 mm × 245 mm × 25 mm, Corning Incorporated, NY, USA) containing sterile plant agar supplemented with FM medium, and randomly divided into four groups: 1) contact water, 2) contact *A. brasilense*, 3) diffusible water, and 4) diffusible *A. brasilense*. For groups 1 & 2, rice seedlings were treated with sterile water or *A. brasilense* suspension (10^8^ cells/ml) applied directly onto the roots, as previously described (Wiggins et al., 2022; Degon et al., 2023; Thomas et al., 2019). The diffusible treatment (groups 3 & 4) was adapted from a study investigating the effects of *A. brasilense* on *Arabidopsis thaliana*, in which the plant roots were physically separated from bacteria (Mendez-Gomez et al., 2021). In the diffusible treatments, a sterile 20 mm × 200 mm strip of 50-micron filter paper (FPR8X11, Filter Paper, Grade 1, United Scientific) was soaked in either sterile water or *A. brasilense* suspension (10^8^ cells/ml) before being placed along the bottom edge of the Square Bioassay Dishes (LXWXH, 245 mm × 245 mm × 25 mm, Corning Incorporated, NY, USA) to avoid any contact with the plant roots (**Supplementary Figure 1A**). Following the treatments, the rice seedlings were allowed to grow in the Percival growth chamber under the same conditions as described earlier. Phenotypic data were collected seven days post-treatment (dpt). All the treatments and plant handling were performed under sterile conditions inside a laminar flow hood.

### Plant mass phenotyping

The rice seedlings from the different treatment groups were assessed for plant mass phenotypes at 7 dpt. Plant root and total mass data were collected from at least three independent experiments, with an average of 17 plants per treatment. The data were analyzed using a one-way ANOVA in RStudio (R Core Team, 2021) across all four treatments (contact water, contact *A brasilense*, diffusible water, and diffusible *A brasilense*). For *post hoc* analysis, Tukey’s HSD tests were performed in RStudio (R Core Team, 2021) to determine differences among the individual treatments.

### PCR-based detection of bacteria

We used a polymerase chain reaction (PCR)-based assay to detect the presence of bacteria on rice roots. Briefly, we extracted DNA from rice root tissues from the different treatments (contact and diffusible) at 7 dpt using the Qiagen Plant DNA extraction kit (Cat # 69104, California, USA), per the manufacturer’s instructions. In these reactions, we used gene-specific primers for the *A. brasilense RecA* and *Sp245p10* genes (McMillan and Pereg, 2014; Soares et al., 2021), and the rice *Cyclophilin* gene primers as a positive control. The PCR products were analyzed by electrophoresis on 2% (w/v) agarose gel and imaged using BIORAD Gel Doc™ EZ Imager. These experiments were performed with at least three biological replicates.

### RNA sequencing (RNA-seq) and data analysis

To identify transcriptomic responses in rice roots in response to diffusible signals from *A. brasilense*, we performed RNA sequencing (RNA-seq). The samples for the RNA-seq experiment included: (a) rice roots + diffusible water; (b) rice roots + diffusible *A. brasilense*. Total RNA was extracted from rice roots at 1 dpt using the Qiagen RNeasy^®^ Plant Mini Kit (Cat #74904, California, USA) per manufacturer’s protocol. The RNA samples were treated with Ambion^®^ DNA-free™ DNase Treatment and Removal kit (Cat #AM1906, California, USA) and assessed for purity (260/280) and yield using a Nanodrop OneC (Thermo Scientific, Delaware, USA) spectrophotometer. The samples were then sent to Novogene Genomics Services and Solutions (CA, USA) for the RNA integrity testing, library preparation, and sequencing. The libraries were pooled and sequenced on the Illumina Novaseq 6000 platform, yielding paired-end reads. The raw sequence data are publicly available from the Sequence Read Archive (SRA) under the BioProject accession ID PRJNA1472649. Statistical analysis of RNA-seq data was performed as in previous studies (Wiggins et al., 2022; Degon et al., 2023; Thomas et al., 2019). Briefly, raw reads (150 base pairs) were processed using *Trimmomatic* (Bolger et al., 2014) version 0.39 to remove Illumina adapter sequences (if any), trim read ends if quality score drops below a specified threshold (Phred score <15), and discard reads shorter than 50 base pairs after all filtering criteria are applied. Paired-end reads were aligned to the rice genome (*Oryza sativa*) using *STAR* version 2.7.9a (Dobin et al., 2013), allowing two mismatches. Alignments were quantified by gene loci and normalized to fragments per kilobase of transcripts per million mapped reads (FPKM) values using *cufflinks* version 2.2.1 (Trapnell et al., 2010). The *Oryza sativa* reference genome (version 7) and gene annotations were obtained from the Rice Genome Annotation Project (Kawahara et al., 2013). Differential expression analysis was performed using *cuffdiff* (a component of Cufflinks), and significant differentially expressed genes (DEGs) were defined as those with Benjamini-Hochberg adjusted P-value or false discovery rate (FDR) <0.05 and absolute fold-change greater than 1.5 (|FC|>1.5). Unexpressed genes or genes with severely low expression were omitted by Cuffdiff.

### Reverse Transcriptase (RT)-PCR experiments

Using reverse transcriptase-polymerase chain reaction (RT-PCR), we validated the expression pattern of seven random selected DEGs identified in the RNA-seq experiment (**Supplementary Table 4**). First, the RNA samples were treated with Ambion^®^ DNA-free DNase Treatment and Removal kit (Cat #AM1906, Foster City, CA, USA) to remove any genomic DNA contamination. Following that, three sets of cDNA were synthesized from 300 ng of total RNA for each treatment using the Thermo Scientific RevertAid RT Kit (#K1691) with Oligo(dT)_18_ primers, per the manufacturer’s instructions. *Cyclophilin* primers were used as the internal reference gene in our analysis. The seven genes and their corresponding primer sequences are included in **Supplementary Table 4**. The primers designed for these genes were based on information on the Rice Genome Annotation Project (https://rice.uga.edu/). The RT-PCR products were visualized via electrophoresis on 2% (w/v) agarose gels. The data shown are representative of three biological replicates per sample.

## Results

### Diffusible signals from *Azospirillum brasilense* promote rice growth

We investigated whether *A. brasilense* could improve rice growth when the plant roots were not in contact with PGPB. We adapted an experimental system from an earlier study with the model plant, *Arabidopsis thaliana* (Mendez-Gomez et al., 2021), in which plant roots and bacteria were physically separated (diffusible treatment). The primary adaptation was an increase in plate size to accommodate rice. We observed that diffusible signals from *A. brasilense* significantly increased root mass in rice plants compared to a similar treatment with water, 7 days post-treatment (dpt) (one-way ANOVA, F_(3,220)_ = 9.451, p < 0.001, Tukey’s HSD, p = 0.02) (**Figure 1A**). Rice root mass also increased significantly upon contact with *A. brasilense* compared to a similar treatment with water (Tukey’s HSD, p = 0.004) (**Figure 1A**). Interestingly, we observed no significant difference (Tukey’s HSD, p = 0.88) in rice root mass with or without contact with *A. brasilense* (**Figure 1A**). No significant difference was also observed in root mass between the contact and diffusible water treatments (Tukey’s HSD, p = 0.93). While root mass showed significant increases in response to diffusible signals, total plant mass showed a non-significant trend (one-way ANOVA, F_(3,220)_ = 5.088, p = 0.002, Tukey’s HSD, p = 0.14) (**Figure 1B**). However, we observed a significant increase in total rice mass when treated directly with *A. brasilense* compared with a similar treatment with water (Tukey’s HSD, p = 0.03) (**Figure 1B**). We also observed no significant differences in total mass between the two treatments, whether with water or *A. brasilense*.

**Figure 1:**
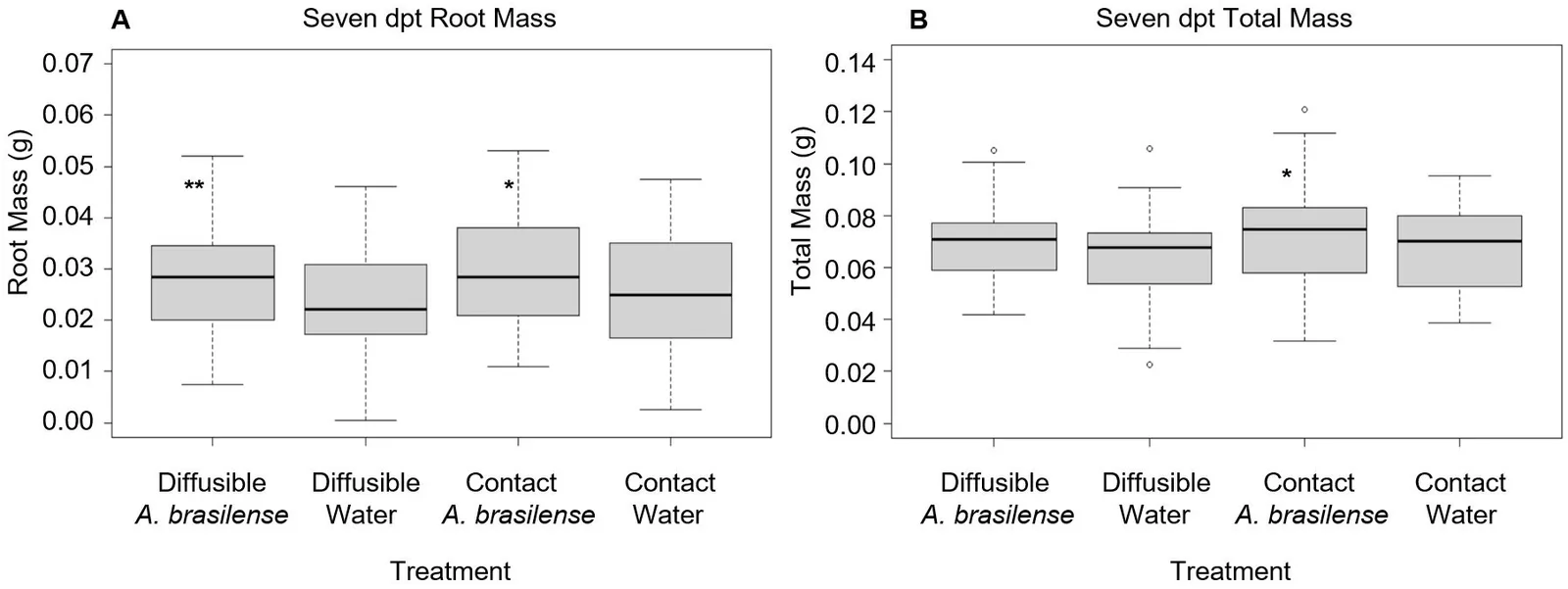
Growth promotion in wild-type rice plants (Nipponbare cv.) by contact and diffusible treatments with *A. brasilense*, seven days post-treatment (dpt). **(A)** Shows that the root mass (g) of wild-type rice plants increased with contact and diffusible treatments with *A. brasilense* compared to the equivalent controls. Asterisk (*) denotes a significant difference compared to the contact water treatment by ANOVA (p < 0.001) and Tukey’s HSD *post hoc* (p = 0.004). Double asterisk (**) denotes a significant difference compared to the diffusible water treatment by ANOVA (p < 0.001) and Tukey’s HSD *post hoc* (p = 0.02). **(B)** Shows that the total mass (g) of wild-type rice plants increased upon contact with *A. brasilense* compared to the equivalent controls. Asterisk (*) denotes a significant difference compared to the contact water treatment by ANOVA (p = 0.002) and Tukey’s HSD *post hoc* (p = 0.03). Open circles indicate outliers. Data represents the average of at least three independent experiments.

To confirm that *A. brasilense* were not in contact with the plant roots in the diffusible treatment, we performed PCR reactions on DNA extracted from rice roots at 7 dpt from both contact and diffusible treatments. In these PCR reactions, we used primers specific to *A. brasilense* genes *RecA* and *Sp245p10* (McMillan and Pereg, 2014; Soares et al., 2021) and rice *Cyclophilin* gene primers as a control. Our results show that *A. brasilense*-specific bands were amplified for both *RecA* and *Sp245p10* genes when the plants were in contact with *A. brasilense* (**Supplementary Figure 1B**). However, we did not observe the expected PCR bands for the bacterial genes when the plants were exposed to the diffusible treatment, despite successful PCR amplification of the plant control gene (**Supplementary Figure 1B**).

### Analysis of rice root transcriptome in response to diffusible signals from *A. brasilense*

Using RNA-seq, we identified differentially expressed genes (DEGs) in rice roots in response to diffusible signals from *A. brasilense* for the following samples: rice (*Oryza sativa* cv. Nipponbare) + water; rice (*Oryza sativa* cv. Nipponbare) + *A. brasilense*. Samples were collected at one-dpt from three biological replicates. RNA sequencing was performed using 150 base pairs, paired-end approach on the Illumina NovaSeq 6000 platform. We obtained an average of 52 million reads per sample with approximately 97% of reads passing the quality control criteria and 95% of these mapping to the rice genome (**Supplementary Table 1**). We observed higher correlations among biological replicates within each treatment group relative to replicates in different groups (**Figure 2A**). Next, DEGs were identified at an adjusted P-value or false discovery rate FDR<0.05 and a fold change FC>1.5. We identified 2516 DEGs in rice roots (**Figure 2B**, **Supplementary Table 2**), including 1766 upregulated and 750 downregulated genes in the treatment group relative to the control group (**Supplementary Table 2**).

**Figure 2:**
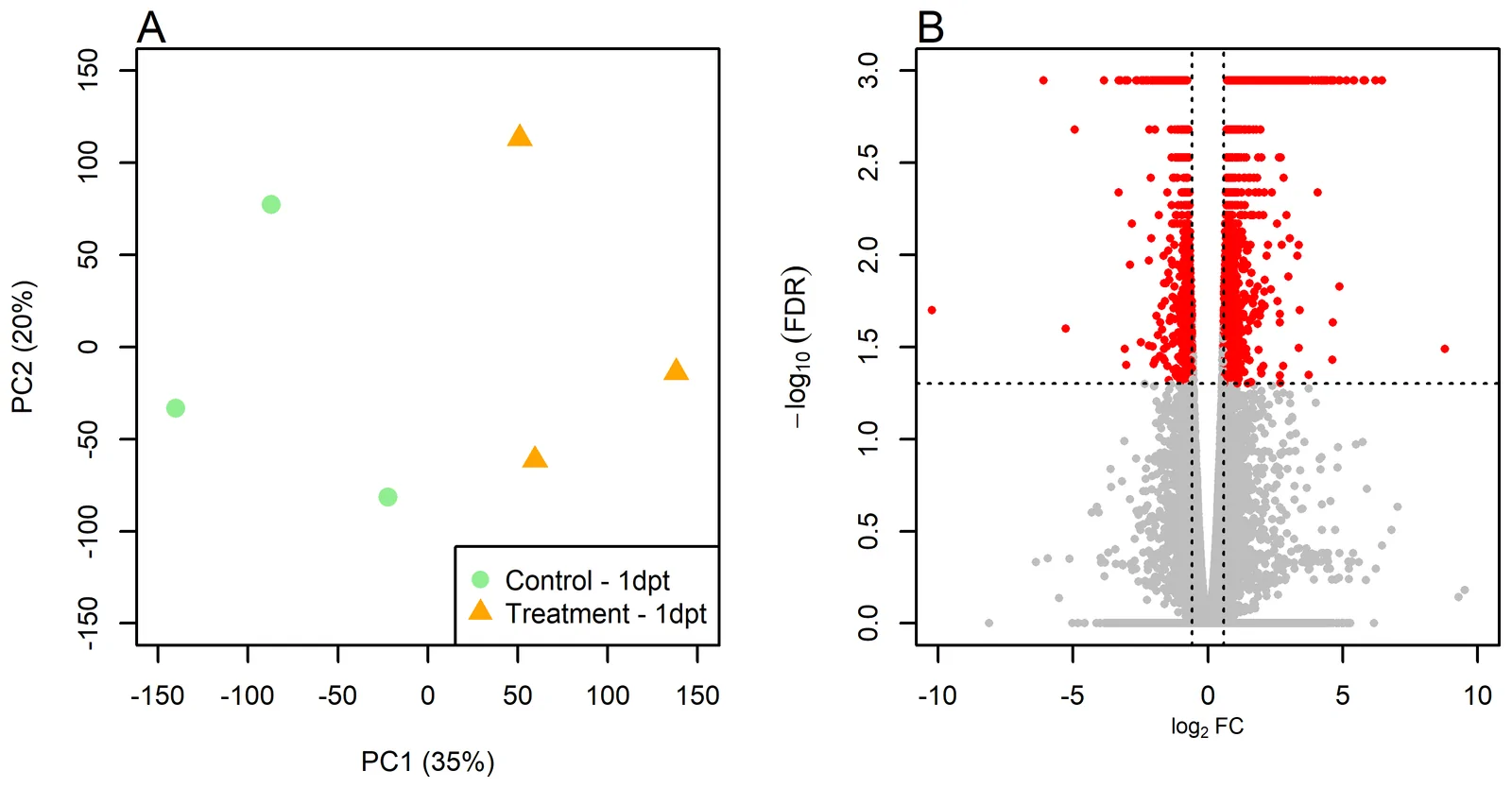
**(A)** PCA plot of RNA-seq samples for the diffusible water treatment and diffusible *A. brasilense* treatment at one-dpt. The plot shows distinct separation of the two treatments. **(B)** Volcano plot of log_2_ fold change plotted against the –log_10_ (FDR) adjusted P-values. Significant DEGs (FDR adjusted P-value < 0.05 and |FC|>1.5) are represented in red color. The non-differentially expressed genes are represented in gray color. Each dot represents one gene.

To understand the biological significance of the 2516 DEGs, we performed a Gene Ontology (GO) analysis using agriGO (Du et al., 2010; Tian et al., 2017) and identified eighty-five significantly over-represented GO terms. There were forty-nine terms in biological processes (e.g., regulation of transcription, oxidation-reduction, regulation of biosynthetic process, response to chemical stimulus, regulation of metabolic process), thirty-three terms in molecular functions (e.g., oxidoreductase activity, antioxidant activity, kinase activity, transcription factor activity, and microtubule motor activity), and three terms in cellular components (extracellular region, apoplast, and ubiquitin ligase complex) (**Figure 3**).

**Figure 3:**
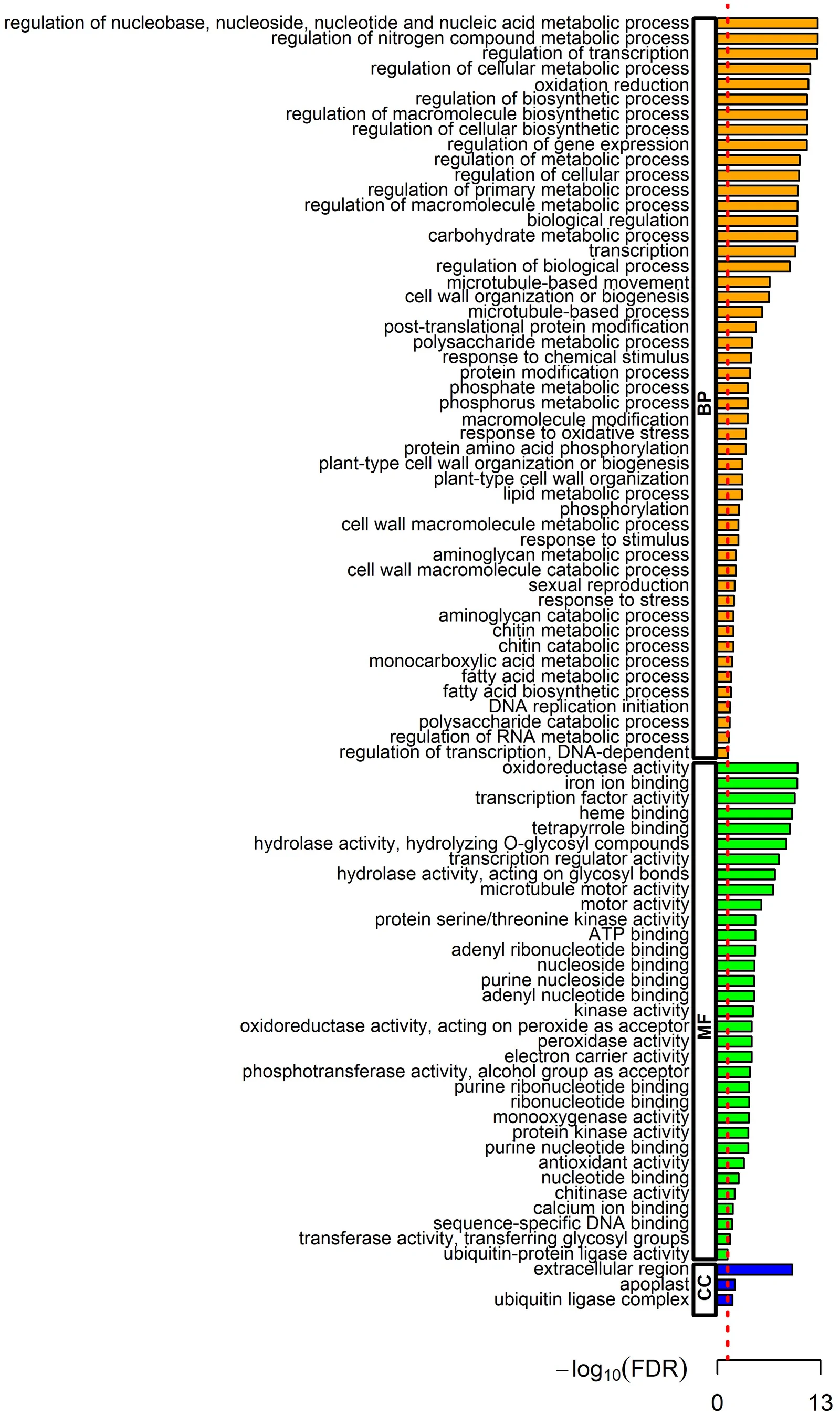
Bar chart showing the top Gene Ontology (GO) terms over-represented in the DEGs. In total, forty-nine terms were identified in biological processes, thirty-three terms in molecular functions, and three terms in cellular components. The X-axis indicates the -log_10_ (FDR) adjusted P-value and the dotted red line indicates the threshold FDR = 0.05. Biological processes (BP), molecular functions (MF), and cellular components (CC) terms are respectively represented by orange, green, and blue bars.

Next, we performed a comprehensive data mining of the DEG list to identify genes potentially involved in the initial signaling between the two partners in this plant-PGPB interaction. We identified several differentially expressed genes from the flavonoid biosynthesis pathway (e.g., chalcone synthase, flavonol synthase) and defense-response genes (e.g., phenylalanine ammonia lyase, thionins, chitinases, pathogenesis-related genes). Among plant receptors, we identified genes encoding Lysin Motif (LysM) domain-containing proteins and a Lysin Motif Receptor-Like Kinase (LYK). We also identified several genes that belong to major plant transcription factor families, including the AP2/ERF (APETALA 2)/ethylene-responsive factor) transcription factors, MYB (myeloblastosis oncogene) family, WRKY family, and GRAS family. Many genes (e.g., auxin-responsive genes, auxin response factors, auxin efflux carriers, and 1-aminocyclopropane-1-carboxylate [ACC] oxidases) involved in hormone signaling were also differentially expressed (**Supplementary Table 2**). Together, these findings suggest a coordinated regulation of gene expression involving defense, secondary metabolism (e.g., flavonoid synthesis genes), signaling, and hormonal pathways in rice roots in response to diffusible signals from *A. brasilense*.

### Comparative transcriptomic analysis of rice roots: with or without contact with *A. brasilense*

Previously, transcriptomic changes in rice roots upon direct application of *A. brasilense* at 1 dpt were reported (Thomas et al., 2019). To better understand the shared and unique transcriptomic responses between contact and diffusible treatments, we compared the DEGs identified in this study with those from the direct treatment dataset (**Supplementary Table 3**). We identified 835 DEGs common to both treatments, 1681 DEGs unique to the diffusible treatment, and 1833 DEGs unique to the contact treatment (**Figure 4A**).

**Figure 4:**
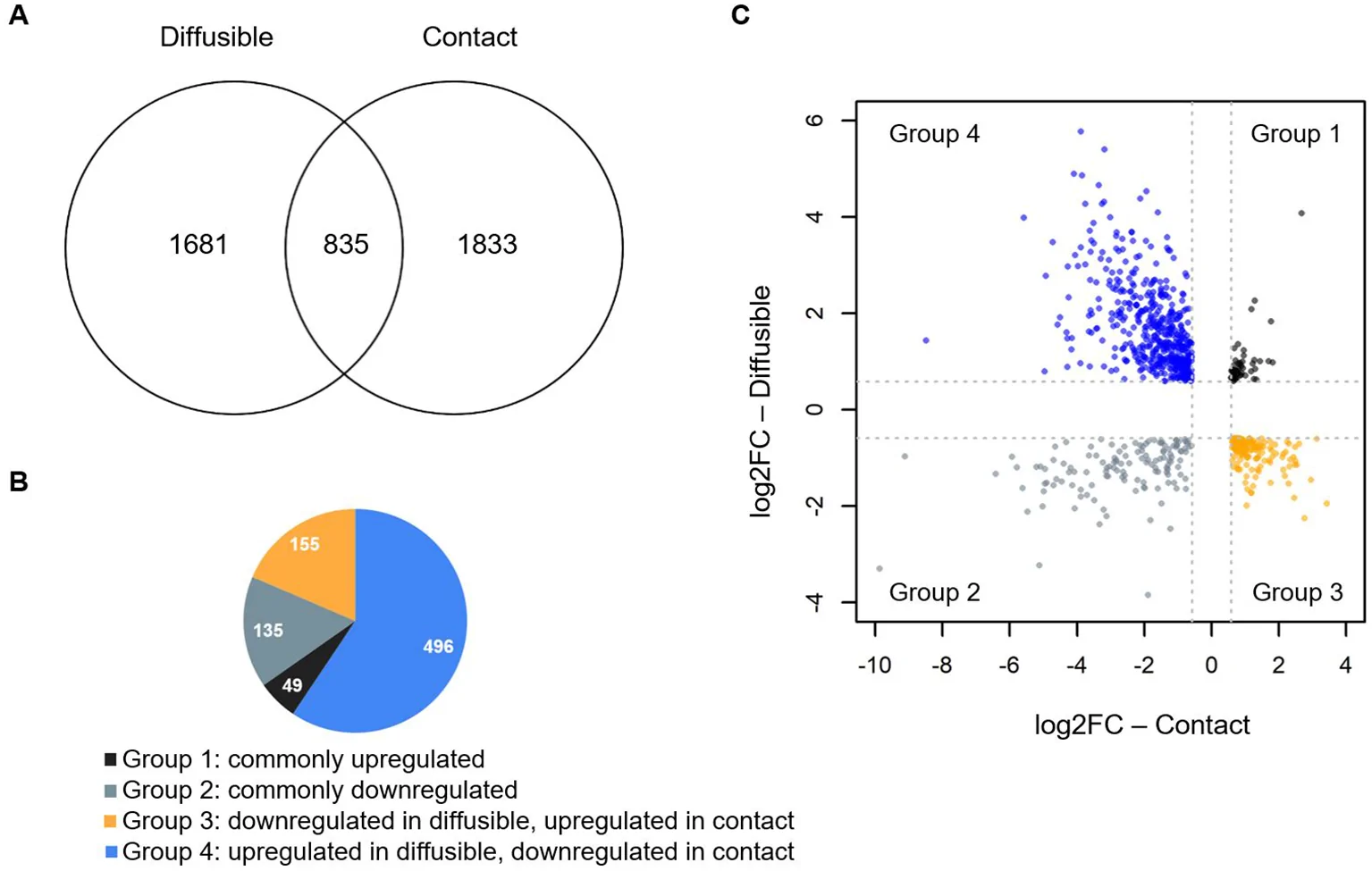
**(A)** Venn diagram showing the DEGs identified in rice roots in response to the contact and diffusible treatments. Eight hundred and thirty-five DEGs were common to both treatments, 1681 DEGs unique to the diffusible treatment, and 1833 DEGs unique to the contact treatment. **(B)** A pie chart showing the distribution of the 835 DEGs common to both contact and diffusible treatments in four different groups. Group 1 (black) includes the commonly upregulated (49 DEGs), Group 2 (gray) includes the commonly downregulated (135 DEGs), Group 3 (orange) includes downregulated in diffusible/upregulated in contact (155 DEGs), and Group 4 (blue) includes downregulated in contact/upregulated in diffusible (496 DEGs). **(C)** Scatter plot showing the fold changes of significantly differentially expressed genes identified in rice roots in response to the contact and diffusible treatments.

#### DEGs common to both treatments

Using GO analysis on the 835 common DEGs, we identified thirty-one terms in biological processes (e.g., regulation of transcription, oxidation reduction, response to chemical stimulus), seventeen terms in molecular functions (e.g., transcription factor activity, oxidoreductase activity, protein serine/threonine kinase activity), and three terms in cellular components (extracellular region, ubiquitin ligase complex, and apoplast) (**Supplementary Figure 2**). Genes encoding flavonol synthase, pathogenesis-related protein, phenylalanine ammonia lyases, chitinases, thionins, transcription factors (e.g., AP2/ERFs, WRKYs, GRAS), and genes involved in hormone signaling were identified in this common group (**Supplementary Table 3**).

The 835 common DEGs can be further divided into four groups based on their distinct expression patterns. Group 1 included 49 genes upregulated in both treatments, Group 2 included 135 genes downregulated in both treatments, Group 3 included 155 genes downregulated in the diffusible treatment but upregulated in the contact treatment, and Group 4 included 496 genes upregulated in the diffusible treatment but downregulated in the contact treatment (**Figures 4B, C**). Notably, almost 78% (651 of 835; Groups 3 & 4) of the common DEGs displayed contrasting expression patterns between the two treatments, with 59.4% of genes (496 of 835; Group 4) upregulated in the diffusible treatment but downregulated in the contact treatment (**Figure 4**). GO analysis of genes in Group 1 identified three over-represented terms in biological processes (response to chemical stimulus, response to stimulus, and response to stress) (**Supplementary Figure 3**), while genes in Group 2 were over-represented with oxidation-reduction (**Supplementary Figure 4**). Genes in Group 3 were over-represented for GO terms associated with oxidation-reduction, response to chemical stimulus, regulation of transcription, and regulation of nitrogen compound metabolic process (**Supplementary Figure 5**). Finally, genes in Group 4 were over-represented with GO terms associated with regulation of transcription, regulation of biosynthetic process, and regulation of nucleobase, nucleoside, nucleotide and nucleic acid metabolic process (**Supplementary Figure 6**).

#### DEGs unique to each treatment

Additionally, we identified 1681 DEGs unique to the diffusible treatment. GO analysis identified thirty-three terms in biological processes (e.g., microtubule-based process, oxidation-reduction, cell wall organization or biogenesis, DNA replication initiation, regulation of transcription), twenty-seven terms in molecular functions (e.g., microtubule motor activity, oxidoreductase activity, kinase activity, transcription factor activity), and one term in cellular components (extracellular region) (**Supplementary Figure 7**). Genes encoding chalcone synthases, a *LYK* receptor, a nodulation-signaling pathway 2 protein (a GRAS transcription factor), and several auxin response and transport genes were unique to the diffusible treatment (**Supplementary Table 3**).

Finally, we identified 1833 DEGs unique to the contact treatment. GO analysis identified thirty-three terms in biological processes (e.g., oxidation reduction, regulation of transcription, regulation of nitrogen compound metabolic process, carbohydrate metabolic process, transport, photosynthesis), twenty terms in molecular functions (e.g., oxidoreductase activity, antioxidant activity, transporter activity, transcription factor activity, protein serine/threonine kinase activity), and five terms in cellular components (e.g., membrane, extracellular region, nucleosome, protein-DNA complex) (**Supplementary Figure 8**). Examples of genes in this group included genes encoding naringenin 2-oxoglutarate 3-dioxygenases, pathogenesis-related protein, *LYK* genes, and nitrate transporters (**Supplementary Table 3**).

### Gene expression validation

Lastly, we validated the expression patterns of seven genes (**Supplementary Table 4**) differentially expressed in response to diffusible signals from *A. brasilense* (**Figure 5**). The genes we tested in the RT-PCR experiments included Early Nodulin 93, a GRAS family transcription factor, a Ser/Thr receptor-like kinase, a probable indole-3-acetic acid-amido synthetase, a kinesin motor domain-containing protein, a histidine kinase, and a LysM domain-containing protein (**Figure 5**). The RT-PCR results confirm the expression pattern of these genes observed in the RNA-seq experiment. The RT-PCR results represent at least three biological replicates.

**Figure 5:**
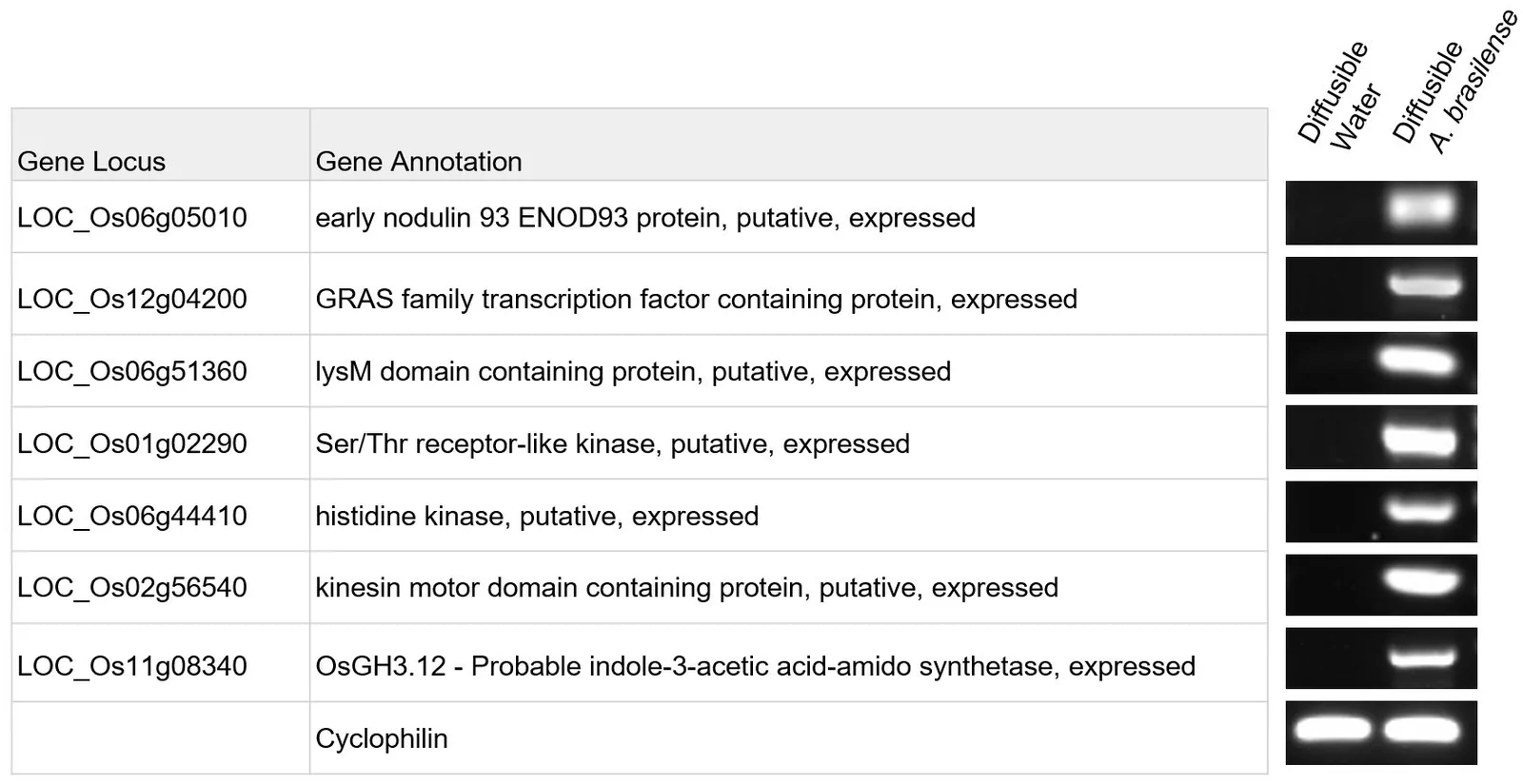
RT-PCR validation of the seven differentially expressed genes (DEGs) identified via RNA sequencing. Diffusible water and diffusible *A. brasilense* represent cDNA templates synthesized from their respective RNA samples. *Cyclophilin* was used as an internal reference gene. RT-PCR was performed in three biological replicates for all samples.

## Discussion

Studies in major plant-microbe symbioses, such as the legume-rhizobia symbiosis and AM symbiosis, have identified the plant (e.g., flavonoids and strigolactones) and microbial signals (Nod- and Myc- factors) required to initiate these interactions (Peters et al., 1986; Maillet et al., 2011; Denarie and Cullimore, 1993; Dénarié et al., 1996; Akiyama and Hayashi, 2006; Besserer et al., 2006). Since most plant-PGPB studies involved contact between plant roots and bacteria, the early signaling mechanisms underlying these interactions are not well defined. More recently, one study reported that diffusible signals from the PGPB *Azospirillum brasilense* could promote growth in the model plant, *Arabidopsis thaliana* (Mendez-Gomez et al., 2021). In this study, we report that diffusible signals from *A. brasilense* are sufficient to promote rice growth and induce significant transcriptional changes in plant roots. Furthermore, a comparative transcriptomic analysis of contact (Thomas et al., 2019) and no-contact (diffusible) treatments with *A. brasilense* revealed both shared and unique gene expression patterns in rice, offering further insights into the underlying transcriptomic changes that regulate this important plant-PGPB interaction.

Using an experimental system suited for rice, we observed that diffusible signals from *A. brasilense* increased root and total mass in rice compared with equivalent water treatments (**Figure 1**). Also, the growth-promotion effects in rice were comparable between the contact and diffusible treatment conditions (**Figure 1**). Next, we confirmed that *A. brasilense* were not present on the plant roots in the diffusible treatment, suggesting that the observed growth promotion was not dependent on contact between *A. brasilense* and rice roots. Our findings corroborate results observed in *A. thaliana* (Mendez-Gomez et al., 2021) and provide evidence that diffusible signals from a PGPB can promote growth in a crop species. Furthermore, our results suggest that the initial growth promotion in rice in response to diffusible signals from *A. brasilense* is comparable to that observed with bacterial contact. However, additional studies are needed to determine whether this effect can be sustained over a longer period or under other growth conditions.

Studies have shown that diffusible signals from plant-beneficial microbes induce morphological, physiological, and transcriptomic responses in the host plant (Jardinaud et al., 2016; Camps et al., 2015; Breakspear et al., 2014; Czaja et al., 2012; Olah et al., 2005; Dénarié et al., 1996; Kosuta et al., 2003; Mukherjee and Ané, 2011a). Using RNA sequencing, we identified a set of differentially expressed genes in rice roots in response to diffusible signals from *A. brasilense*. Several of these genes may regulate plant responses and signaling pathways, including flavonoid synthesis genes, defense-response genes, genes encoding receptors and transcription factors, and genes involved in hormone signaling. Below, we discuss some of these findings.

### Genes involved in flavonoid biosynthesis

Studies on LRS and AM symbiosis have shown that flavonoids are secreted from host plant roots in the presence of their symbiotic microbial partners and trigger the molecular dialogue between them (Abdel-Lateif et al., 2012; Hassan and Mathesius, 2012; Peters et al., 1986). Some studies have reported that flavonoids promote the colonization of plant roots by PGPB (Webster et al., 1998; Gough et al., 1997). Interestingly, recent transcriptomic studies in rice have identified differential expression of genes involved in flavonoid synthesis, suggesting a possible role for flavonoids in plant-PGPB interactions (Brusamarello-Santos et al., 2012; Wiggins et al., 2022; Thomas et al., 2019). In this study, we identified several DEGs (e.g., chalcone synthases and flavonol synthases) involved in flavonoid synthesis in rice roots. Chalcone synthase (CHS) is a key enzyme in the biosynthesis of chalcones, one of the precursors in flavonoid biosynthesis (Rudrapal et al., 2021; Wang et al., 2025). Genetic studies in *M. truncatula* showed that silencing of *CHS* affected root nodulation (Wasson et al., 2006; Zhang et al., 2009). *CHS* genes were also upregulated upon treatment with Rhizobium sp. NGR234 and its Nod factors in *Vigna unguiculata* (Krause et al., 1997), and by *S. meliloti* (Ng et al., 2015; Breakspear et al., 2014) and its Nod factors in *M. truncatula* (Breakspear et al., 2014). During AM symbiosis, *CHS* genes were induced in the roots of *M. truncatula* during the initial stages of the interaction (Bonanomi et al., 2001), and upon treatment with Myc factors (Camps et al., 2015), and in onion plants upon inoculation with mycorrhiza (Mollavali et al., 2017). We identified several *CHS* genes (e.g., LOC_Os05g12180, LOC_Os05g12240, LOC_Os10g08620, LOC_Os05g12210) upregulated in rice in response to diffusible signals from *A. brasilense*. *CHS* genes were also differentially expressed when *A. brasilense* were in contact with rice roots (Thomas et al., 2019). The flavonol synthase (FLS) gene encodes a key enzyme in the flavonoid biosynthetic pathway (Wang et al., 2025; Holton et al., 1993). The *FLS* gene was expressed in *M. truncatula* roots by rhizobia (Ng et al., 2015), roots and nodules of the actinorhizal plant, *Datisca glomerata* (Gifford et al., 2018), and in onion upon mycorrhizal inoculation (Mollavali et al., 2017). We identified two rice *FLS* genes (LOC_Os02g52840 and LOC_Os09g18450) upregulated in response to diffusible signals from *A. brasilense.* A few *FLS* genes (e.g., LOC_Os01g61610, LOC_Os03g03034) were also upregulated upon contact with *A. brasilense* (Thomas et al., 2019). Another important plant-derived flavonoid molecule that plays a key role in plant-microbe interactions is naringenin. Its role in mediating the LRS is well documented (Begum et al., 2001; Ng et al., 2015), but one study also reported that naringenin promoted wheat root colonization by the PGPB, *Azorhizobium caulinodans* (Webster et al., 1998). Although not induced by diffusible signals from *A. brasilense*, we noted that two naringenin genes (LOC_Os04g49194 and LOC_Os04g49210) were upregulated in rice roots in contact with the PGPB (Thomas et al., 2019). The expression patterns of these flavonoid biosynthesis genes in host plant roots in response to beneficial microbes and their signals appear broadly conserved across plant-microbe symbioses. Further studies should investigate their precise role in plant roots throughout the rice- *A. brasilense* interaction.

### Defense-response genes

Studies have shown that the host plant modulates its defense responses during interactions with beneficial microbes (Soto et al., 2009; Liang et al., 2013; Toth and Stacey, 2015). For example, some well-characterized plant defense genes (e.g., phenylalanine ammonia-lyase [*PAL*], chitinases) are transiently induced during the early stages of LRS, but their expression is suppressed as the interaction progresses to facilitate accommodating their symbiotic microbial partner inside the root tissues (Breakspear et al., 2014; Kouchi et al., 2004; Soto et al., 2009; Brechenmacher et al., 2008). While a reduction in gene expression in some defense genes (e.g., *PAL*, chitinases, thionins) has also been observed in host plants when in contact with different PGPB, their expression pattern when the plant roots are not in contact with bacteria is not known (Wiggins et al., 2022; Thomas et al., 2019; Brusamarello-Santos et al., 2012). In this study, we identified certain *PAL* (e.g., LOC_Os02g41670, LOC_Os05g35290, LOC_Os02g41680), chitinase (e.g., LOC_Os05g33140, LOC_Os02g39330, LOC_Os04g41680), and thionin (e.g., LOC_Os06g31800, LOC_Os06g31280) genes upregulated by diffusible signals from *A. brasilense.* Interestingly, these genes were downregulated when rice was in contact with *A. brasilense* (Thomas et al., 2019). The expression patterns of these genes follow a similar transient induction pattern observed during LRS. Further studies can confirm whether these transcriptomic responses are related to regulating the extent of root colonization by *A. brasilense* or are required for the progression of the interaction.

Pathogenesis-related (*PR*) genes are a major class of plant defense genes that play important roles during interactions with pathogenic and beneficial microbes. Some *PR* genes are upregulated in rice upon exposure to *Magnaporthe oryzae*, a pathogen (Kawahara et al., 2012). However, during interactions with beneficial microbes, including PGPB, some *PR* genes are downregulated in their host plants (Brusamarello-Santos et al., 2012; Wiggins et al., 2022; Thomas et al., 2019; Peleg-Grossman et al., 2012; Chen et al., 2017). Here, we identified several *PR* genes (e.g., LOC_Os08g28670, LOC_Os12g36880, LOC_Os12g36840) that were downregulated in rice roots under both *A. brasilense* treatments, suggesting that their expression likely remains suppressed in rice throughout the interaction.

### Receptors

We identified several genes (e.g., Lysin Motif [LysM] domain-containing proteins, Lysin Motif Receptor-Like Kinases [LYK]) associated with signal perception in the rice root transcriptome in response to diffusible signals from *A. brasilense*. Besides playing crucial roles in the perception of symbiotic microbial signals during LRS and AM symbiosis, the LysM domain-containing receptors are also involved in mediating defense responses during plant-microbe interactions (Jones and Dangl, 2006; Bozsoki et al., 2020, 2017; Amor et al., 2003; Gough and Cullimore, 2011; Rey et al., 2013; Madsen et al., 2003; Radutoiu et al., 2003; Limpens et al., 2003; Miyata et al., 2014). We identified several LysM domain-containing genes (LOC_Os06g51360, LOC_Os03g01990, and LOC_Os09g37600) upregulated in expression in rice roots in response to *A. brasilense* under diffusible treatment conditions. Genes encoding LysM domain-containing proteins (LOC_Os06g51360, LOC_Os11g34570, and LOC_Os10g38040) were also differentially expressed in rice when *A. brasilense* were directly applied onto plant roots (Thomas et al., 2019). One subclass of the LysM-RLK family playing key roles in plant-microbe interactions is the LYK receptor family. These perceive Nod factors and serve as entry receptors in LRS (Limpens et al., 2003; Smit et al., 2007), and also play a dual role in plant immunity and symbiosis with AM fungi (Gibelin-Viala et al., 2019). *LYK* genes have been shown to be upregulated by purified Nod factors in *M. truncatula* (Jardinaud et al., 2016). The rice *OsLYK7* (LOC_Os11g35330) was upregulated in response to diffusible signals from *A. brasilense*, but was not differentially expressed in roots in contact with *A. brasilense* (Thomas et al., 2019). Interestingly, two genes encoding *LYK* receptors, *OsLYK1* (LOC_Os02g09960) and *OsLYK2/OsNFR5* (LOC_Os03g13080), were differentially expressed in rice roots in contact with *A. brasilense* (Thomas et al., 2019), but not expressed when the bacteria were not in contact with plant roots. Since these receptors have already been shown to play multiple roles in interactions with both symbiotic and pathogenic microbes, further investigations can determine whether they are involved in plant-PGPB interactions.

### Transcription factors

Genetic studies in plant-microbe interactions have identified the roles of diverse families of transcription factors (e.g., AP2-ethylene response factor (ERF), WRKYs, MYB, GRAS) during these interactions (Yano et al., 2017; Diedhiou and Diouf, 2018; Ripodas et al., 2014; Kalo et al., 2005; Heckmann et al., 2006; Chakraborty et al., 2022). Some of these TFs are also differentially expressed upon application of Nod factors on plant roots (Rose et al., 2012; Camps et al., 2015). Interestingly, a few transcriptomic studies on plant-PGPB interactions have also reported differential expression of some of these transcription factors, suggesting their possible involvement in these interactions (Wiggins et al., 2022; Thomas et al., 2019; Hardoim et al., 2020; Brusamarello-Santos et al., 2012). In this study, we identified a number of different transcription factor families (e.g., AP2/ERF, MYB, WRKY) differentially regulated in rice roots in response to diffusible signals from *A. brasilense*. Interestingly, while some of these genes (e.g., LOC_Os02g45420 [*AP2*], LOC_Os01g46800 [*WRKY*], LOC_Os01g52410 [*MYB*]) were upregulated in expression in the diffusible treatment, they were downregulated in rice roots upon contact (Thomas et al., 2019). The induction of these transcription factors in response to diffusible signals from *A. brasilense* suggests that these genes may be involved during the initial stages of the *rice-A. brasilense* interaction. Their downregulation in plant roots in contact with *A. brasilense* also suggests that this suppression may be necessary to facilitate the progression of the interaction.

Another key family of transcription factors involved in beneficial plant-microbe interactions is the GRAS family, including the nodulation signaling pathway proteins 1 & 2 (NSP1 and NSP2) (Ripodas et al., 2014; Heckmann et al., 2006; Kalo et al., 2005). Previously, the rice homologs of NSP1 and NSP2 were shown to fully rescue the nodulation-deficient phenotypes of the *nsp1* and *nsp2* mutants in *L. japonicus*, suggesting a conservation of functions of these genes in a non-legume plant like rice (Yokota et al., 2010). A recent study in rice reported that phosphorus starvation induces expression of *NSP1* and *NSP2*, which regulate transcription of genes involved in strigolactone biosynthesis and Myc factor recognition (Hong et al., 2026). We identified several GRAS-family transcription factors (e.g., LOC_Os12g04200, LOC_Os11g04400, LOC_Os03g51330) upregulated in response to the diffusible signals from *A. brasilense*, including the gene (LOC_Os03g15680) encoding NSP2 (**Supplementary Table 3**). However, when the plant roots were in contact with *A. brasilense*, these genes were either downregulated or not differentially expressed (Thomas et al., 2019). Understanding the involvement of these genes and their specific role(s) in the rice-*A. brasilense* interaction will require advanced studies.

### Hormone-related genes and nitrate transporters

Since PGPB improve plant growth via hormone synthesis and improved nutrient uptake, it is reasonable to expect genes involved in hormone signaling pathways and nutrient uptake (especially nitrate transporters) to be differentially expressed in the host plants during the interaction. Several transcriptomic studies identified differential expression of genes from these classes during interactions with different PGPB (Wiggins et al., 2022; Thomas et al., 2019; Camilios-Neto et al., 2014; Brusamarello-Santos et al., 2012; Rekha et al., 2018; Hardoim et al., 2020). Auxin is a major plant hormone implicated in diverse roles, including beneficial plant-microbe interactions (Mathesius et al., 1998; Mathesius, 2008; Mukherjee and Ané, 2011b). Some studies reported differential expression of genes involved in auxin signaling upon application of Nod factors (Breakspear et al., 2014; Rose et al., 2012). In this study, we identified several genes involved in auxin response and transport, including *SAUR* genes (e.g., LOC_Os01g56240, LOC_Os04g51890), Auxin Response Factors (e.g., LOC_Os04g56850, LOC_Os06g09660), Auxin-responsive *Aux/IAA* genes (e.g., LOC_Os02g56120, LOC_Os03g43410), *GH3* (LOC_Os11g08340 and LOC_Os07g40290), Auxin Efflux Carriers (e.g., LOC_Os01g45550, LOC_Os06g44970) upregulated in response to diffusible signals from *A. brasilense*. However, most of these genes were downregulated (e.g., LOC_Os01g56240, LOC_Os04g56850, LOC_Os02g56120, LOC_Os04g56850, LOC_Os01g45550) or not differentially expressed (e.g., LOC_Os04g51890, LOC_Os06g09660, LOC_Os03g43410, LOC_Os07g40290, LOC_Os06g44970) upon contact with *A. brasilense* (**Supplementary Table 3**). Additionally, VOCs from *A. brasilense* and other PGPBs have been reported to promote plant growth by modulating the auxin signaling pathway in host plants (Mendez-Gomez et al., 2021; Ye et al., 2025; He et al., 2023). Ethylene is another major plant hormone that regulates diverse plant processes, including plant-microbe interactions (Olanrewaju et al., 2017; Nascimento et al., 2018; Mukherjee and Ané, 2011b). Here, we identified a gene encoding the 1-aminocyclopropane-1-carboxylate oxidase (ACC) protein (LOC_Os09g27820), a precursor in the ethylene biosynthesis pathway, which was upregulated in response to diffusible signals from *A. brasilense*. However, this gene, along with several other ACC-encoding genes, was downregulated when *A. brasilense* was in contact with plant roots (Thomas et al., 2019). Several ACC-encoding genes were also downregulated in rice and wheat when in contact with *H. seropedicae* and *A. brasilense*, respectively (Brusamarello-Santos et al., 2012; Camilios-Neto et al., 2014). Taken together, the expression pattern of these auxin- and ethylene-related genes suggests an initial induction of these hormonal pathways in response to diffusible signals followed by suppression when rice roots are in contact with *A. brasilense*.

Nitrate transporters are important proteins that enable plants to efficiently take up nitrate ions, transport them from roots to shoots, and support plant growth (Pellizzaro et al., 2017; Fan et al., 2017). High-affinity nitrate transporters (NRTs) are key genes involved in nitrogen acquisition and distribution in plants (Bagchi et al., 2012; Sigalas et al., 2023; Luo and Nan, 2024). Transcriptomic studies identified some NRTs (LOC_Os02g38230 and LOC_Os01g50820) upregulated in rice roots in contact with *A. brasilense* and *H. seropedicae* (Wiggins et al., 2022; Thomas et al., 2019). Interestingly, none of these transporters were differentially expressed in response to diffusible signals from *A. brasilense*, suggesting that activation of these plant nutrient transporters is a downstream process and likely requires contact between the plant roots and bacteria.

## Summary

To summarize, we report that diffusible signals from *Azospirillum brasilense* can promote growth in a crop species, rice, even in the absence of contact between the bacterium and plant roots. Using RNA sequencing, we identified major gene expression trends in rice roots without bacterial contact, including differential expression of genes involved in flavonoid biosynthesis, defense responses, receptors, transcription factors, and hormones. We also observed downregulation of some *pathogenesis-related* genes and no differential expression of nitrate transporters. Furthermore, a comparative transcriptomic analysis of diffusible and contact treatments with *A. brasilense* identified shared and unique gene expression trends (**Table 1**). For example, some flavonoid biosynthesis genes were upregulated under both treatments, suggesting a possible role of flavonoids throughout the interaction. In contrast, some *PR* genes were suppressed under both treatments, suggesting the plant adjusts its immunity to facilitate its interaction with *A. brasilense*. Interestingly, certain defense-response genes (*PAL*, chitinase, thionin), transcription factors (AP2/ERFs, MYBs, WRKYs, and GRAS), and hormone-related (auxin and ethylene) genes were induced in diffusible treatment, but downregulated or not differentially expressed in rice roots in contact with bacteria, implying the initial induction and subsequent suppression of these genes may be crucial for establishing the interaction with *A. brasilense*. Also, the absence of differential expression of nitrate transporters in the diffusible treatment, but their expression in rice roots in contact with bacteria, suggests their possible involvement at later stages of the interaction. Overall, our findings offer novel insights into gene expression trends in rice roots during the early phase of interaction with *A. brasilense*, before any contact occurs between the two partners. Future studies can focus on characterizing the transcriptomic responses at additional time points, defining the host genetic pathway mediating the response to these signals, and identifying the chemical nature of the diffusible signals.

**Table 1:** Summary of gene expression trends in rice roots in response to diffusible and contact treatments with *Azospirillum brasilense*.

	Diffusible signals	Contact
Flavonoid biosynthesis		
*Chalcone Synthase*	Up	–
*Flavonol Synthase*	Up	Up
*Naringenin*	–	Up
Defense-response		
*PAL, Chitinase,Thionin*	Up	Down
*PR*	Down	Down
Receptors		
*LysM*	Up	–
*LYK*	Up	–
Transcription factors		
*AP2/ERF, MYB, WRKY*	Up	Down
*GRAS*	Up	Down or Not Expressed
Hormone-related		
*Auxin*	Up	Down or Not Expressed
*Ethylene*	Up	Down
Nitrate transporters	Not Expressed	Up

Up indicates upregulation, Down indicates downregulation, and ‘-‘ indicates no observable gene expression trends.

## Data Availability

The datasets presented in this study can be found in online repositories. The names of the repository/repositories and accession number(s) can be found in the article/supplementary material. The raw sequence data are publicly available from the Sequence Read Archive (SRA) under the BioProject accession ID PRJNA1472649.

## Supplementary material

Supplementary Figure 1 (**A**) A representative image of the experimental system used in the diffusible treatments. In these treatments, sterile filter paper was soaked in either sterile water or *A. brasilense* suspension and positioned along the bottom edge of the bioassay dishes. (**B**) Gel electrophoresis image confirming the presence of *A. brasilense* in the contact treatment samples and the absence of bacteria in the diffusible treatment samples. PCR was used to amplify segments of genes, *RecA* and *Sp245p10*, specific to *A. brasilense*. The rice *Cyclophilin* gene was used as a control in this experiment. The PCR products were analyzed via electrophoresis on a 2% (w/v) agarose gel and is a representation of at least three biological replicates. One hundred base pair DNA ladder was used as a reference.

Supplementary Figure 2 Bar chart showing the top Gene Ontology (GO) terms over-represented in the list of 835 DEGs common between diffusible and contact treatments. The X-axis indicates the -log_10_ (FDR) adjusted P-value and the dotted red line indicates the threshold FDR = 0.05. Biological processes (BP), molecular functions (MF), and cellular components (CC) terms are respectively represented by orange, green, and blue bars.

Supplementary Figure 3 Bar chart showing the top Gene Ontology (GO) terms over-represented in the list of 49 upregulated DEGs in both diffusible and contact treatments. The X-axis indicates the -log_10_ (FDR) adjusted P-value and the dotted red line indicates the threshold FDR = 0.05. Biological processes (BP), molecular functions (MF), and cellular components (CC) terms are respectively represented by orange, green, and blue bars.

Supplementary Figure 4 Bar chart showing the top Gene Ontology (GO) terms over-represented in the list of 135 downregulated DEGs in both diffusible and contact treatments. The X-axis indicates the -log_10_ (FDR) adjusted P-value and the dotted red line indicates the threshold FDR = 0.05. Biological processes (BP), molecular functions (MF), and cellular components (CC) terms are respectively represented by orange, green, and blue bars.

Supplementary Figure 5 Bar chart showing the top Gene Ontology (GO) terms over-represented in the list of 155 DEGs that are downregulated in the diffusible treatment but upregulated in the contact treatment. The X-axis indicates the -log_10_ (FDR) adjusted P-value and the dotted red line indicates the threshold FDR = 0.05. Biological processes (BP), molecular functions (MF), and cellular components (CC) terms are respectively represented by orange, green, and blue bars.

Supplementary Figure 6 Bar chart showing the top Gene Ontology (GO) terms over-represented in the list of 496 DEGs that are upregulated in the diffusible treatment but upregulated in the contact treatment. The X-axis indicates the -log_10_ (FDR) adjusted P-value and the dotted red line indicates the threshold FDR = 0.05. Biological processes (BP), molecular functions (MF), and cellular components (CC) terms are respectively represented by orange, green, and blue bars.

Supplementary Figure 7 Bar chart showing the top Gene Ontology (GO) terms over-represented in the 1681 DEGs unique to the diffusible treatment. The X-axis indicates the -log_10_ (FDR) adjusted P-value and the dotted red line indicates the threshold FDR = 0.05. Biological processes (BP), molecular functions (MF), and cellular components (CC) terms are respectively represented by orange, green, and blue bars.

Supplementary Figure 8 Bar chart showing the top Gene Ontology (GO) terms over-represented in the 1833 DEGs unique to the contact treatment. The X-axis indicates the -log_10_ (FDR) adjusted P-value and the dotted red line indicates the threshold FDR = 0.05. Biological processes (BP), molecular functions (MF), and cellular components (CC) terms are respectively represented by orange, green, and blue bars.

Supplementary Table 1 Summary of raw reads, the number of reads that survived QC criteria, and reads that were successfully mapped to the rice genome. There were three biological replicates of diffusible water treatment (control11, control12, control15) and diffusible *A. brasilense* treatment (treatment11, treatment14, treatment15).

Supplementary Table 2 List of the differentially expressed genes (DEGs) in rice roots in response to diffusible signals from *A. brasilense*, one day post treatment. The table contains gene identifiers, gene annotations, nominal P-values, adjusted P-values, and log_2_ fold change values.

Supplementary Table 3 List of the differentially expressed genes (DEGs) in rice roots with or without contact with *A. brasilense*, one day post treatment. The table contains a list of the DEGs whose expression is common to both treatments or unique to each treatment with their annotations, gene IDs, and their corresponding log_2_ fold change values in the diffusible and contact treatments.

Supplementary Table 4 List of the forward and reverse primer sequences of the genes used in the RT-PCR experiments.
